# Growing Evidence of Exosomal MicroRNA-Related Metastasis of Hepatocellular Carcinoma

**DOI:** 10.1155/2020/4501454

**Published:** 2020-11-27

**Authors:** Wenbing Sun, Shuqi Fu, Size Wu, Rong Tu

**Affiliations:** Department of Medical Imaging, The First Affiliated Hospital of Hainan Medical University, Haikou, Hainan 570102, China

## Abstract

Metastasis is the prominent cause of death in patients with hepatocellular carcinoma (HCC); however, the mechanisms behind HCC metastasis are not well understood. MicroRNAs (miRs) can regulate gene expression and affect HCC metastasis. Exosomes can transport miRs and other cargoes to and from different cells, thus being associated with tumour-distant metastasis. Exosomal miRs involve different processes of HCC metastasis through their functional effects, such as their induction of epithelial-to-mesenchymal transition, angiogenesis, and distant niche. In this review, data from the literature were analysed and summarised, with a focus on the evidence extraction of exosomal miRs in HCC metastasis with the purpose of increasing the understanding of the mechanisms behind HCC metastasis and acquiring implications for application.

## 1. Introduction

Hepatocellular carcinoma (HCC) is the fourth leading cause of cancer-associated death and the sixth incidence of malignancy worldwide, accounting for ~780,000 deaths and 840,000 new cases in 2018 [1]. Currently, the 5-year survival rate of HCC is 5–30%, and the causes of poor prognosis are due to delayed diagnosis, metastasis, and recurrence [2–5]. Malignant cells metastasizing to distant locations of the body are the main cause of cancer-related death. Metastasis has been thought to be a process of “metastatic cascade” where malignant cells infiltrate distant tissue, evade immune defences, adapt to supportive niches, survive as latent tumour-initiating seeds, and eventually break out to replace the host tissue [6]. However, the mechanism behind cancer metastasis is not well understood at the molecular level. Communications between malignant cells and normal cells are indispensable for distant metastatic colonisation of malignant cells, in which microRNAs (miRs) play important roles [7–24].

Exosomes are small extracellular vesicles (EVs) that are released from cells on the fusion of an intermediate endocytic compartment, the multivesicular body, with the plasma membrane [25]. Exosomes carry many cargoes that have various biological functions, including miRs [25]. Tumourigenesis, advancement, and metastasis of malignant tumours are closely associated with exosomes [9, 10, 26]. The HCC tumour microenvironment is composed mainly of fibroblasts, immune cells, and an extracellular matrix, and an abnormal tumour microenvironment is closely associated with HCC metastasis [27, 28]. Studies on exosomal miRs derived from HCC cells and their microenvironment and their roles in HCC metastasis have been increasing, indicating that further understanding of exosomal miRs in HCC is important for timely diagnosis and treatment. In this review, studies on various cell-derived exosomal miRs in HCC are summarised, with a focus on their roles in HCC metastasis.

## 2. Expression of miRs and Cancer

miRs are evolutionarily conserved, endogenous, noncoding single-stranded RNA molecules of 18–25 nucleotides. miRs effectively regulate gene expression by inhibiting the translation of target mRNAs [10]. It is estimated that miRs influence ≥30% of human gene expression, making them one of the largest categories of gene regulators [11]. miR dysregulation is associated with different pathological processes in various tissue types, especially regarding the development of cancer [10, 12]. Furthermore, miRs may be either tumour suppressive or oncogenic, and a number of studies have reported aberrant miR expression in different tumours, including HCC [10, 13–15]. The molecular functional links between abnormal miR expression and malignant transformation are largely reflected by abnormal cellular proliferation and differentiation, angiogenesis, invasion, and metastasis [10, 16]. miRs can be downregulated or absent in cancer, and examples of this include miR-142 [17], miR-424-5p [18], and miR-634 [19]. On the contrary, oncogenic miRs, including miR-192-5p [20], miR-1251-5p [21], and miR-210 [22], are overexpressed in tumours. The mechanisms of specific miRs involving cancer metastasis have been investigated intensively. For example, miR-429 regulates HCC metastasis and epithelial-to-mesenchymal transformation (EMT) by targeting Ras-related protein Rab-23 [23] and miR-145 directly targets the coding sequence of the Golgi membrane protein 1 gene, leading to the inhibition of tumourigenesis and metastasis [24].

## 3. Exosomal miRs and HCC Metastasis

**Figure 1:**
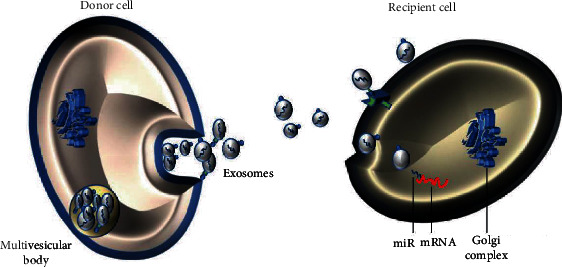
Schematic illustration of miR transfer between cells mediated by exosomes. miR: microRNA.

**Figure 2:**
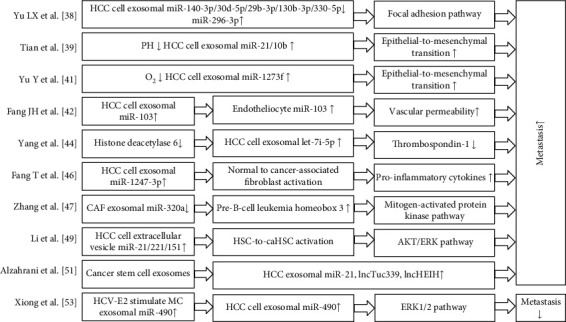
Concise summary of exosomal miRs and HCC metastasis. miR: microRNA; HCC: hepatocellular carcinoma; CAF: cancer-associated fibroblast; HCV: hepatitis C virus; MC: mast cell; HSC: hepatic stellate cell; caHSC: cancer-associated hepatic stellate cell.

Exosomes are small EVs (diameter, 30-100 nm) released by various cell types and rich in functional molecules such as lipids, proteins, mRNA, and miRs [25, 29]. Exosomes possess surface molecules that allow them to target recipient cells and subsequently transport their contents to the recipient cytoplasm through the following mechanisms: (i) receptor-ligand interaction to induce signal transmission, (ii) endocytosis and/or phagocytic internalisation, and (iii) fusion with the target cell membrane [30].  Figure 1 illustrates exosome transport of miRs. These miRs have been shown to exert functional effects on the recipient cell, directly modulating their mRNA targets to create a premetastatic environment suitable for tumour cell colonisation and growth [31, 32]. Many studies have reported a close relationship between HCC metastasis and aberrant miR expression, such as miR-365, which can target disintegrins and metalloproteinase 10; the low expression of miR-365 is significantly associated with tumour node metastasis stage and lymph node metastasis, and the overexpression of miR-365 significantly inhibits cell proliferation, colony formation, migration, and invasion of HCC [33]; miR-203a-3p.1 acts as an onco-miRNA targeting the IL-24 gene of a tumour suppressor. When it is downregulated and expressed, it promotes HCC growth and metastasis [34]. Unprotected miRs in the blood are easily and rapidly degraded, while exosomal miRs are not affected by endogenous ribonucleases [35]. Previous studies have shown that a specific repertoire of integrins is expressed on tumour-derived exosomes, which dictates exosome adhesion to specific cell types and extracellular matrix molecules, particularly organs, so the organotrophic integrins of exosomes are favoured in the cargo transported to target cells, thus leading to the determination of organotrophic metastasis by tumour exosomal integrins [36]. Therefore, the levels of exosomal miR can be used to more accurately evaluate the association between miRs and HCC metastasis compared with those of nonexosomal miRs. The study of miRs and their related functional mechanisms in exosomes may contribute to the diagnosis and treatment of HCC metastasis. Hepatic stellate cells (HSCs), fibroblasts, cancer stem cells (CSCs), and mast cells (MCs) in the HCC microenvironment are also involved in HCC metastasis, in addition to the exosomal miRs derived from HCC cells. The relationship between exosomal miRs secreted by various cell types and HCC metastasis is summarised in Figure 2. In the literature, some investigators describe the vesicles that deliver miRs using the term EVs, but their description of the methods of extraction and identification are the same as those of exosomes. Indeed, EVs also contain exosomes, so EVs are regarded as the same entity as exosomes and are included in the current review.

### 3.1. Exosomal miRs Derived from HCC Cells

Focal adhesion kinase (FAK) has been reported as being associated with cancerogenesis and the development of HCC, and miRs may modulate cancer cell migration and metastasis by influencing FAK-facilitated cytoskeletal rearrangement, as well as stroma adhesion reconstruction [37]. For example, in the study by Yu et al. [38], fresh HCC tissues obtained from surgical resection were cultured; exosomes were extracted from the HCC cells using a 3D medicine exosome isolation kit, and exosomal miRs were detected through miRNA-Seq analysis. The results showed the decreased expression of five miRs associated with strong migrating ability in wound-healing assays (miR-140-3p, miR-30d-5p, miR-29b-3p, miR-130b-3p, and miR-330-5p), as well as the increased expression of miR-296-3p. These miRs are also enriched in the “focal adhesion” pathway. An investigation of the association between miR expression level and survival rate further verified that miRs can mediate HCC metastasis.

Tian et al. [39] reported that exosomal miR-21 and miR-10b can increase in acidic conditions and can promote the growth and metastasis of HCC; the experiment showed that miR-21 and miR-10b increased in exosomes isolated from HCC cells that were cultured in acidic conditions. If these miRs transferred to HCC cells that were cultured under normal conditions, it could enhance HCC cell invasion and migration abilities; this experiment was validated in xenograft mouse models. The high expression levels of miR-21 and miR-10b in exosomes promoted HCC metastasis to the lungs. In HCC patients, serum exosomal miR-21 and miR-10b levels were found to be significantly increased and positively correlated with the clinical stage when compared with those of healthy volunteers.

A hypoxic tumoural microenvironment can induce angiogenesis and proliferation of tumour [40, 41]. In a study by Yu et al. [41], exosomes were extracted from the culture medium of HCC cells by ultracentrifugation. The quantity of exosomes and exosomal miR-1273f was significantly increased in HCC cells cultured under hypoxic conditions and was effectively transferred to HCC cells cultured under normoxic conditions. The HCC cells showed increased proliferation, invasiveness, migration, and subsequent EMT. Following the use of an miR-1273f antagonist, the above situation was reversed, indicating that increased miR-1273f expression promotes the metastasis of HCC.

Blood-borne metastasis is the primary cause of mortality in patients with cancer and increased vascular permeability facilitates metastasis [36]. A study by Fang et al. [42] showed that miR-103 secreted from HCC cells could be delivered to endothelial cells by exosomes, preventing the expression of human vascular endothelial cadherin, zonula occludens 1, and p120-catenin protein; weakening endothelial connectivity integrity; increasing vascular permeability; and accelerating metastasis. A xenograft mouse model with high miR-103 expression exhibited a higher probability of intrahepatic and pulmonary metastasis. Furthermore, HCC patients with high serum expression levels of miR-103 possessed a higher metastasis potential than patients with low miR-103 expression levels; therefore, these data indicate that the expression of exosomal miR-103 is positively associated with HCC metastasis.

Histone deacetylase 6 (HDAC6) has a crucial role in multiple cellular processes, and the dysregulation of HDAC6 may be associated with the progression of some cancers [43]. In the ambient microenvironment, HCC cells can induce HCC-like characteristics in untransformed hepatocytes via exosomal let-7i-5p transfer. Moreover, the deletion or inhibition of HDAC6 decreases thrombospondin-1 translation in a let-7i-5p-associated manner, thus disrupting the regulation of thrombospondin-1 protein in the growth, proliferation, angiogenesis, and metastasis of HCC [44].

### 3.2. Exosomal miRs Derived from HCC Cells and Cancer-Associated Fibroblasts (CAFs)

As the most prolific cell type in the cancer microenvironment, CAFs are a subset of transformational fibroblasts that facilitate tumour progression and metastasis [45]. Fang et al. [46] reported that exosomal miR-1247-3p derived from HCC cells activated the *β*1-integrin-NF-*κ*B signalling pathway by downregulating *β*-1,4-galactosyltransferase III, thus transforming fibroblasts into CAFs. CAFs promoted metastasis by secreting IL-6 and IL-8 and proinflammatory cytokines that enhanced stemness and EMT in HCC cells. In addition, the serum exosomal miR-1247-3p levels in HCC patients with lung metastases were notably more elevated than those in patients without lung metastases. These data suggest that HCC cell-derived exosomal miR-1247-3p can promote an inflammatory microenvironment and lung metastasis.

In a study by Zhang et al. [47], CAFs and paracancer fibroblasts (PAFs) were taken from HCC tumour tissues and cultured, and exosomes were isolated from the culture medium and analysed. Compared with those from PAFs, CAF-derived exosomes exhibited lower levels of miR-320a, which can directly target Pre-B-cell leukaemia homeobox 3 and inhibit the activation of the mitogen-activated protein kinase signalling pathway, thereby suppressing metastasis. This indicates that decreased levels of miR-320a may facilitate HCC metastasis.

### 3.3. Exosomal miRs Derived from HSCs

HSCs are the major interstitial cell type in the HCC microenvironment and an important component of invasion and metastasis [48]. A study by Li et al. [49] showed that EV-associated miR-21, miR-221, and miR-151 secreted by HCC cells could activate the transformation of HSCs into cancer-associated HSCs with a malignant phenotype; in turn, the EV-associated secretion of these miRs by cancer-associated HSCs enhanced the propagation and incursion of HCC cells. Collectively, these phenomena promote HCC metastasis, which was verified by experiments in xenograft animals.

### 3.4. Exosomes Derived from CSCs and HCC-Derived Exosomal miR

CSCs may be the result of the malignant transformation of normal stem cells that have stronger self-renewal ability and metastatic potential [50, 51], and exosomes from CSCs are important mediators of chemotherapy resistance and tumour metastasis. In a study by Alzahrani et al. [51], exosomes were extracted from CSCs and injected into mice, which were induced with HCC using diethylnitrosamine. Compared with mice injected with PBS, the quantity and size of cancer lesions were boosted; the results showed that exosomal miR-21, lncRNA Tuc339, and HCC upregulated EZH2-associated lncRNA (derived from HCC) were all highly expressed, along with a variety of other active molecules. Collectively, these aberrations reduced apoptosis, increased angiogenesis, and induced EMT to increase the incidence of metastasis.

### 3.5. Exosomal miRs Derived from MCs

Studies have shown that MCs are abundant in the cancer microenvironment [52]. For example, Xiong et al. [53] reported that after stimulated MCs with the hepatitis C virus E2 envelope glycoprotein, MC-derived exosomal miR-490 was highly expressed, and the miR-490 level in HCC cells was increased following coculture with exosomes derived from MCs, subsequently decreasing the migrating ability of HCC cells. The use of miR-490 inhibitors augmented the migratory capacity, and the exosome transfer of miR-490 from MCs to HCC cells was demonstrated as being able to inhibit HCC metastasis.

## 4. Conclusions

Exosomes are critical mediators of intercellular communication, through which miRs are transported between various different cells. The high expression of oncomiRs and low expression of tumour suppressing miRs can result in the formation of metastatic foci. The occurrence of metastasis depends not only on exosomal miRs secreted by HCC cells but also on a variety of other cells in the HCC microenvironment. In the HCC microenvironment, by transferring exosomal miRs, HCC cells can activate the conversion of fibroblasts, HSCs, and even normal liver cells into cancer-related cells, resulting in malignant phenotypes. This can also weaken the integrity of endothelial cell connectivity and increase vascular permeability. In turn, exosomal miRs derived from activated HSCs, fibroblasts, and CSCs can promote HCC metastasis.

HCC metastasis is accompanied by the abnormal expression of exosomal miRs, and the detection of this aberrant expression can be used to evaluate HCC metastasis and prognosis. Exosomal miRs that promote or inhibit HCC metastasis may be used for prevention and treatment. Tian et al. [39] developed a nanotargeted drug for the therapeutic target of HCC, which can reduce the expression of exosomal miR-21 and miR-10b that may promote the metastasis of HCC; and experiments with animal models have demonstrated a significant reduction in tumour growth and lung metastasis foci of HCC.

After more than two decades of research, great progress has been made in the field of exosomal biology; however, it is still considered to be in its infancy, and the role of exosomes in tumour progression requires further investigation. Advancement in understanding of exosomal miRs will make them become promising candidates for the diagnosis and treatment of HCC.
